# Multi-tumor analysis of cancer-stroma interactomes of patient-derived xenografts unveils the unique homeostatic process in renal cell carcinomas

**DOI:** 10.1016/j.isci.2021.103322

**Published:** 2021-10-21

**Authors:** Kuniyo Sueyoshi, Daisuke Komura, Hiroto Katoh, Asami Yamamoto, Takumi Onoyama, Tsuyoshi Chijiwa, Takayuki Isagawa, Mariko Tanaka, Hiroshi Suemizu, Masato Nakamura, Yohei Miyagi, Hiroyuki Aburatani, Shumpei Ishikawa

**Affiliations:** 1 Department of Preventive Medicine, Graduate School of Medicine, The University of Tokyo, Experimental Research Buliding, 12^th^Floor, 7-3-1, Hongo, Bunkyo-ku, Tokyo 113-8654, Japan; 2 Department of Thoracic Surgery, Tokyo Medical and Dental University, Yushima, Bunkyo-ku, Tokyo 113-8519, Japan; 3 Division of Gastroenterology and Nephrology, Department of Multidisciplinary Internal Medicine, School of Medicine, Faculty of Medicine, Tottori University, Tottori 683-8504, Japan; 4 Central Institute for Experimental Animals, Tonomachi, Kawasaki-ku, Kawasaki, Kanagawa 210–0821, Japan; 5 Data Science Center, Jichi Medical University, Yakushiji, Shimotsuke-shi, Tochigi 329–0498, Japan; 6 Department of Pathology, Graduate School of Medicine, The University of Tokyo, Tokyo 113–8654, Japan; 7 Department of Regenerative Medicine, Tokai University School of Medicine, Shimokasuya, Isehara, Kanagawa 259–1193, Japan; 8 Research Institute, Kanagawa Cancer Center, Nakao, Asahi-ku, Yokohama 241–8515, Japan; 9 Division of Genome Sciences, RCAST, The University of Tokyo, Tokyo 113–8654, Japan

**Keywords:** Cancer systems biology, Cancer

## Abstract

The patient-derived xenograft (PDX) model is a versatile tool used to study the tumor microenvironment (TME). However, limited studies have described multi-tumor PDX screening strategies to detect hub regulators during cancer-stroma interaction. Transcriptomes of cancer (human) and stroma (mouse) components of 70 PDX samples comprising 9 distinctive tumor types were analyzed in this study. PDX models recapitulated the original tumors' features, including tumor composition and putative signaling. Particularly, kidney renal clear cell carcinoma (KIRC) stood out, with altered hypoxia-related pathways and a high proportion of endothelial cells in the TME. Furthermore, an integrated analysis conducted to predict paracrine effectors in the KIRC cancer-to-stroma communication detected well-established soluble factors responsible for the hypoxia-related reaction and the so-far unestablished soluble factor, apelin (APLN). Subsequent experiments also supported the potential role of APLN in KIRC tumor progression. Therefore, this paper hereby provides an analytical workflow to find hub regulators in cancer-stroma interactions.

## Highlights

• Analysis of multi-tumor PDXs discloses hub regulators in cancer-stroma interplay
• PDX transcriptomes reproduced original tumor's biological characteristics
• Kidney cancer (KIRC) TME is featured with altered hypoxia-related reactions
• Apelin is documented as a potential paracrine effector in KIRC-to-stroma interplay

## Graphical abstract

**Figure:**
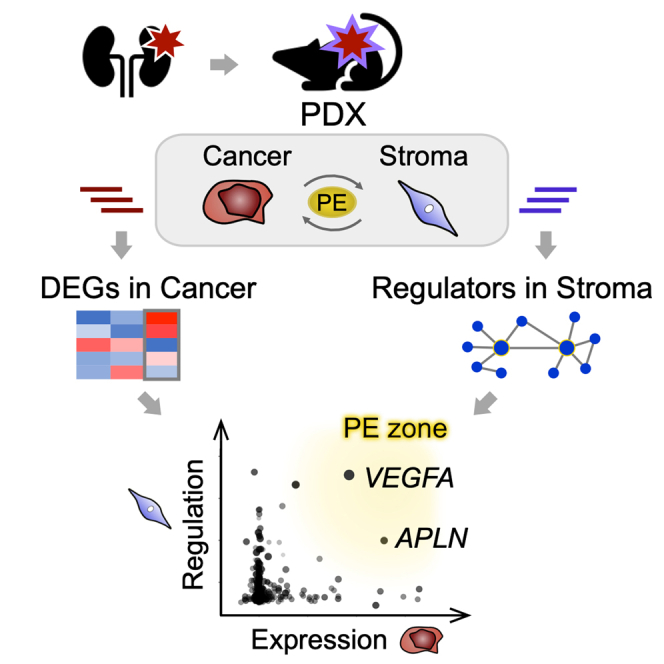

## Introduction

The “seed and soil” hypothesis was first propounded in 1889 to explain the organ preference of cancer metastasis (Paget, 1889). Since then, many studies have elucidated that malignant cell behaviors, including engraftment, growth, invasion, and metastasis, depend on the biological interaction of cancer cells called “seeds,” and the tumor microenvironment (TME), called “soil.” The TME consists of the extracellular matrix (ECM) and host stromal cells, such as endothelial cells, fibroblasts, or immune cells. Malignant cells also induce a disturbance in stromal signaling and metabolism via humoral factor secretion or cell-cell contact. In contrast, the affected stromal cells either have a niche supply for cancer cells by generating ECM or possess neo-vasculature tissues that potentially prevent anticancer agents from permeating the TME or enhance the survival of cancer cells (Hanahan and Weinberg, 2011) (Lyssiotis and Kimmelman, 2017). Therefore, pharmacological interventions to cancer-stroma interactions have been proven as effective therapeutic strategies (Casey et al., 2015) (Valkenburg et al., 2018), thereby highlighting the importance of understanding the TME.

Currently, several methodologies investigate comprehensive cancer-stroma interactomes. Single-cell RNA sequencing (scRNASeq), coupled with tissue dissociation and mass cytometry, has enabled the profiling of TME transcriptomes at the cellular resolution (Helmink et al., 2020) (Goveia et al., 2020). Furthermore, laser capture microdissection (LCM) followed by RNA sequencing, which targets tissue areas of interest to be clipped out by laser under a microscope, is another option. LCM has advantages in being free from the cell dissociation procedure and is aware of histopathological heterogeneity (Civita et al., 2019).

The patient-derived xenograft (PDX) mouse model is thus an alternative tool for TME studies, which is established by the direct engraftment of surgically dissected human cancer fragments into immunologically compromised mice. Xenografted cancers mostly retain microenvironment features, intra-tumor heterogeneity, and transcriptional patterns of original cancer (Choi et al., 2014). Therefore, these biological characteristics enable the wide use of PDX models in drug screening or co-clinical trials (Gao et al., 2015). Also, given that the difference in ortholog sequences between humans and mice can be as high as 15% on average (Waterston et al., 2002), the simultaneous quantification of mixed transcripts is feasible by cross-referencing to each transcript-map (Bradford et al., 2013). Additionally, the model's fidelity and cancer-stroma distinguishability allows researchers to implement comprehensive interactome analyses. Thus, although PDX models lack the sufficient involvement of immune cells in tumors, unlike scRNASeq or LCM methods, the pathophysiological robustness of passage procedures where grown xenografts are re-xenografted to the next generation mice (Gao et al., 2015) gives PDX models several practical superiorities. For example, passages from a small portion of resected specimens can be used as biological replicates in experimental analyses; it is not easy for many lab teams to access fresh materials removed from patients, especially in the case of studies related to epidemiologically rare tumors. Another strong point is that researchers can conduct subsequent preclinical intervention tests on PDX mice of descendant generations after they find the TME signal of interest via interactome analysis.

Pan-cancer omics studies can therefore help expand our perspective concerning malignancies and provide cues to decipher the associated biological dynamics of tumors, as shown in a recent whole-genome analysis on The Cancer Genome Atlas (TCGA) data (Campbell et al., 2020). Based on the transcriptome analysis of PDXs, however, only a few studies have been conducted to characterize the TME of multiple PDX types so far (Bradford et al., 2016). Moreover, most PDX TME studies lacked a step to model the interaction of signal-sending and signal-receiving cells. Instead, the conventional analytical workflow includes a simple differential analysis of stromal transcripts between the two conditions (e.g., a high-grade tumor versus a low-grade tumor), with subsequent pathway analysis to interpret the output in a biological context. Indeed, this approach is practical in describing the signaling alteration in stromal transcriptomes caused by the underlying changes in cancer-stroma interplay. However, such practice possesses a limited capability to clarify causal regulators of the crosstalk, which are potential druggable targets, due to the lack of integrating regulatory network information in the analysis.

Thus, in this study, a collection of 70 PDX samples obtained from tumors of 43 patients and comprising nine distinctive original tumor types were analyzed. To search for master regulators expressed by cancer cells that govern the stromal transcriptome, an integrated analysis of (1) an upstream regulator estimation of the stromal transcriptome, and (2) a differential analysis of the complementary cancer transcriptome was conducted based on the hypothesis that such regulators' expression was promoted in cancer cells. Through the investigation, unique profiles of the kidney renal clear cell carcinoma (KIRC) interactome were found, involving several paracrine molecules represented by vascular endothelial growth factor A (*VEGFA*). In particular, a soluble regulator that has been less recognized in KIRC pathophysiology was identified. Subsequent experiments suggested that the regulator could possess a function associated with KIRC progression in a stroma-dependent manner.

## Results

### Strategic concept to detect hub genes in cancer to stroma interaction

**Figure 1:**
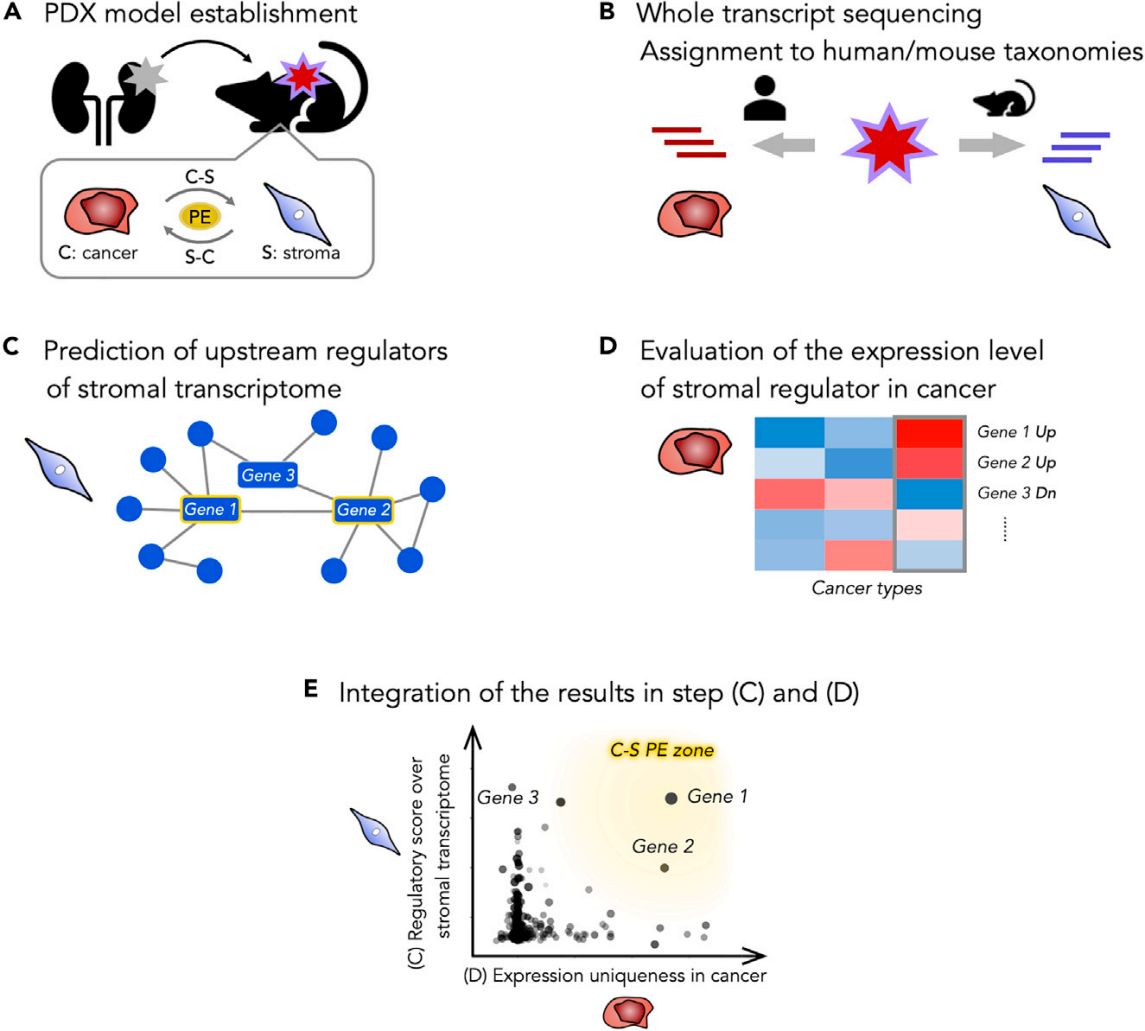
A conceptual illustration of the workflow to detect hub genes during cancer-stroma interaction The pipeline consists of the following five steps. (A): Patient-derived xenograft (PDX) model establishment. Here, 70 PDXs comprising nine different primary tumor types (COAD, NSCLC, EWS, PAAD, KIRC, STAC, GBM, GIST, and BRCA. See the Results section for abbreviation), were established. **PE**: Paracrine effectors. (B): Transcript quantification and assignment to human/mouse taxonomies. Sequenced reads were quantified with reference to the combined transcriptome of human and mouse, then subsequently divided into the right taxonomy, representing cancer cell-derived (human) or stromal cell-derived (mouse) counts, respectively. (C): Prediction of the upstream regulators of the stromal transcriptome. After a differential analysis conducted on the stroma of the PDX types of interest (e.g., the KIRC PDX model vs. the others), the upstream regulators over the detected differentially expressed (DE) genes were estimated using a directional pathway analysis (Ingenuity Upstream Regulator Analysis, IPA). (D): Evaluation of the expression uniqueness of the predicted stromal regulator homologs in the complementary cancer cells. The differential analysis of the complementary cancer transcriptome (e.g., the KIRC PDX model versus the others) yielded a list of DE genes in cancer cells. (E): Integration of the results of steps (C) and (D). The intersection of genes provided in steps (C) and (D) was depicted, with the scores of regulatory effects on the stromal transcriptome and those of the expression uniqueness in the cancer cells, in a scatterplot. The transcripts of the genes located in the right upper area (C-S PE zone) in the plot were considered more as paracrine effectors in the “cancer to stroma” interaction.

The conceptual workflow to predict “cancer to stromal” interaction is depicted in Figure 1. This strategy identified hub effectors, such as soluble factors or cell adhesion molecules uniquely expressed in cancer cells and consequently controlled the transcriptome of stromal cells. The pipeline comprised five steps. Step (A): PDX model establishment. Step (B): Transcript quantification and assignment to human/mouse taxonomies. Step (C): General prediction of upstream regulators of the stromal transcriptome (mouse) using preceding differential analysis on stromal transcripts in the PDX tumor types of interest. Step (D): Evaluation of the expression uniqueness of identified stromal regulator homologs in the complementary cancer cell transcriptome (human). Step (E): Integration of the results of steps (C) and (D).

### The landscape of the patient-derived xenograft transcriptomes

Seventy PDX samples obtained from tumors of 43 patients, and comprising nine distinctive PDX tumor types, were enrolled in this study (Table S1). Samples included 29 colorectal carcinomas (COAD) obtained from 20 patients, 5 non-small cell lung cancers (NSCLC) from 5 patients, 8 Ewing's sarcomas (EWS) from 1 patient, 8 pancreatic ductal adenocarcinomas (PAAD) from 8 patients, 4 KIRCs from 2 patients, 5 stomach adenocarcinomas (STAD) from 2 patients, 4 glioblastomas (GBM) from 1 patient, 4 gastrointestinal stromal tumors (GIST) from 2 patients, and 3 breast invasive carcinomas (BRCA) from 2 patients. The mapping rate of sequenced reads to the combined human and mouse transcriptome was 88.7% on average across all samples, ranging between 79.3% and 94.7%. The mixed transcripts and their count estimates were subsequently sorted into either human or mouse transcript groups, representing cancer or stromal cell-derived count estimates, respectively. With the gene-level filtering and logarithm-transformation, the expression distributions of the identified 17,666 cancer and 17,616 stromal genes were generally equivalent among PDXs. However, they were still skewed due to the substantial number of genes with low expression values in some samples (Figure S1).

**Figure 2:**
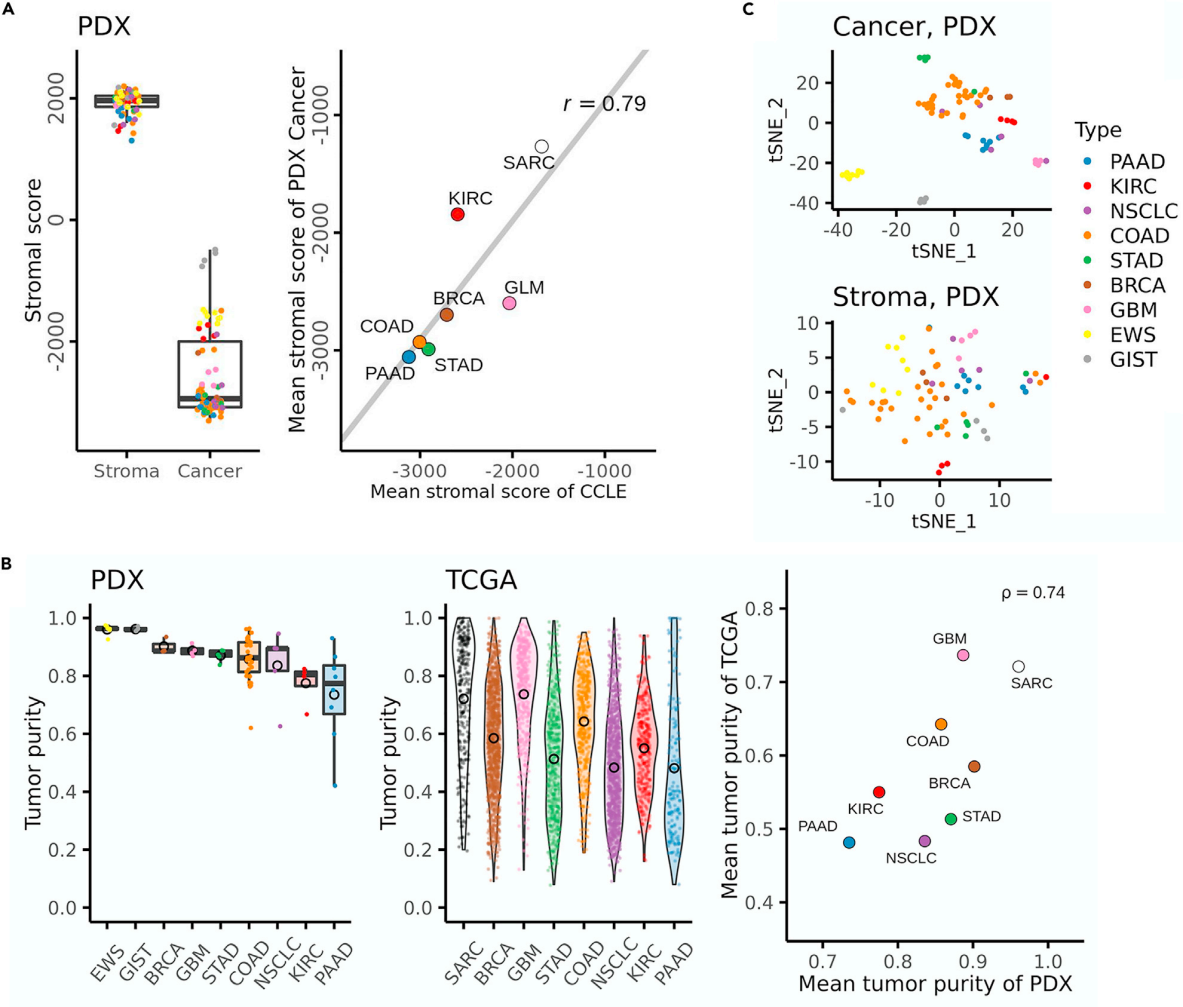
The landscape of the transcriptome of patient-derived xenograft models (A) The ESTIMATE stromal scores of stroma-assigned reads and cancer-assigned reads of PDXs (the left panel). The scatterplot showed mean stromal scores of PDX cancer components and corresponding cancer cell lines (CCLE) as references (the right panel). CCLE included PAAD (n = 41), COAD (n = 58), STAD (n = 41), BRCA (n = 56), KIRC (n = 25), GLM (65 gliomas corresponding to GBM in PDX), SARC (40 sarcomas corresponding to EWS/GIST in PDX). A linear regression line in gray and Pearson's correlation coefficient *r* are shown. (B) Tumor purity of PDXs (the left panel) and non-xenografted cancers (TCGA) as a reference (the middle panel). The PDX tumor purity was defined as the ratio of the cancer gene-level count sum to the cancer plus stroma gene-level count sum. The TCGA tumor purity was computed using the ABSOLUTE algorithm. The scatterplot (the right panel) showed mean tumor purity values of PDX and TCGA, corresponding to small circles in black on the left and middle panels, respectively. The SARC in TCGA corresponds to the EWS and GIST in PDX. Spearman's correlation coefficient ρ is shown. (C) The t-SNE plot of PDXs. Samples of the cancer component (the upper panel) or the stroma component (the lower panel) of the PDXs were plotted in t-SNE format, based on their transcriptome data, with a perplexity value of five. The colors of the dots were used to represent the PDX types as annotated. See also Table S1 and Figure S1.

First, the effectiveness of the read assignment procedure to the human or mouse transcriptome was evaluated by calculating ESTIMATE (Estimation of STromal and Immune cells in MAlignant Cancer tissues using Expression data) (Yoshihara et al., 2013) stromal scores. The stromal scores, which roughly reflected the extent of resemblance to the general expression profiles of the tumor stroma, were conspicuously higher in mouse-derived reads than in human-derived reads in all PDX tumor types (Figure 2A) as expected. Although the scores of PDX cancer reads varied slightly according to tumor types, the tendency of PDX cancer components was generally consistent with that of the corresponding cancer cell lines of the Cancer Cell Line Encyclopedia (CCLE) transcriptome data (Ghandi et al., 2019) (Pearson's correlation coefficient *r* = 0.79, *p*< 0.05; Figure 2A). Next, the relative abundance of cancer and stroma components in PDX tumors was assessed to find possible features specific to tumor types. The purity of cancer-derived transcripts of PAAD or KIRC was markedly lower than that of EWS, GIST, and GBM (*q*< 0.05; Mann-Whitney U test; Benjamini-Hochberg, BH, adjusted), suggesting that the PAAD or KIRC were stromal component-rich tumors (Figure 2B). However, the tumor purity of PDXs (transcripts-based) was overall higher than that of the non-engrafted tumor samples obtained from TCGA data (CNV-based) (Figure 2B). Still, the relative stroma-abundance across PDX tumor types was similar in those two cohorts (the Spearman's rank correlation coefficient ρ = 0.74, *p*< 0.05), with the EWS and the GIST grouped to correspond to the TCGA sarcoma (SARC).

### Tumor type-dependent characteristics of the PDX TMEs

The t-SNE projection disclosed that the cancer cells' expression profiles were similar within each PDX tumor type (Figure 2C), except for the NSCLC samples, which included various histological types; three adenocarcinomas, one squamous cell carcinoma, and one poorly differentiated non-small carcinoma of undefined subtypes. Concerning stromal expression data, alternatively, dimension reduction procedures or clustering methods revealed no evident biologically understandable results (Figure 2C). This result indicated two possibilities; (1) PDX models could not reproduce tumor stroma, and (2) PDX models reproduced tumor stroma to some extent, but the underlying inter-tumor-type variance in the stroma was minor compared to the intra-tumor-type variance or other non-specific variances. Given the successful separation of most PDX cancers by tumor type, it is conceivable that potential cancer-stroma interactions could induce tumor type-specific signaling in the corresponding stroma. It is also important to note that the intra-tumor-type variances in stroma should be intrinsically more significant than cancer cells in light of the polyclonality, diversity, and redundancy of the stroma cell population. Therefore, we decided to explore possibility (2) above by investigating the expression patterns of curated gene sets in the stroma to focus on biologically meaningful expression variances.

**Figure 3:**
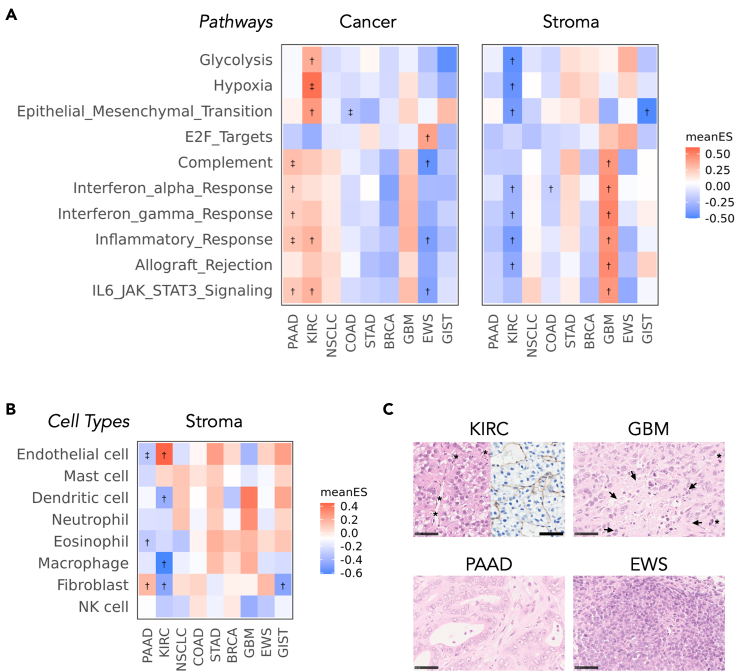
Tumor type-dependent characteristics of the PDX TMEs (A) Pathway analysis. The GSVA analysis was conducted for 40 of 50 hallmark pathways. The top 10 pathways measured to be statistically significant (*q*< 0.005, BH adjusted) using the one-way ANOVA test for PDX-types were shown. (B) Cell type-specific gene set analysis. The GSVA analysis was conducted for gene sets specific to the eight major stromal cell types. The mean enrichment scores (meanES) of each PDX type, scaled to the sample-wise standard deviation (SD) and divided by the total SD, were shown by color scales. †q<0.05; ‡q<0.005(BH adjusted). (C) Pathological images of the KIRC, PAAD, GBM, and EWS PDXs on hematoxylin and eosin (H&E) stain. The right half of the KIRC picture was stained using the anti-CD31 antibody. Asterisks (∗) denote intra-tumor vessels in the KIRC and mitotic tumor cells in the GBM. Arrows in the GBM show necrotic areas infiltrated by inflammatory cells. Scale bars represent 50 μm in length. See also Tables S2 and S3.

The GeneSet Variation Analysis (GSVA) (Hänzelmann et al., 2013) was conducted using the 40 Hallmark biological pathways (Table S2) of the Molecular Signatures Database (Liberzon et al., 2015) and eight stromal cell types (Table S3) (Schelker et al., 2017). The heatmap of the mean enrichment scores of the top 10 pathways in the analysis of stromal transcriptomes (*q*< 0.005, one-way ANOVA test for PDX-types, BH adjusted) showed upregulated inflammatory or interferon (IFN)-related pathways in the GBM stroma (Figure 3A). This finding was in accordance with the fact that expressions of dendric cells- or neutrophil-specific markers were relatively promoted in the GBM stroma, albeit statistically insignificant (Figure 3B). As for the histopathological aspect, GBM PDXs were confirmed to be composed of pleomorphic tumor cells with irregular-shaped nuclei and sporadic necrotic tissues infiltrated by inflammatory cells (Figure 3C). Concerning PAAD, a fibroblast-rich stroma was implied by enrichment analysis (Figure 3B). Then hematoxylin and eosin (H&E) staining of PAAD PDXs revealed the pathological features that tumor cells surrounded by abundant fibrotic tissue were arranged in a tubular form (Figure 3C). Alternatively, EWS PDXs, a representative of high tumor purity PDXs (Figure 2B), were microscopically featured as sheets of round cancer cells with a high nuclear-cytoplasm ratio, having poor interjacent stroma (Figure 3C). Collectively, PDXs were confirmed to maintain the original TME architecture; besides, the underlying pathophysiology of the tumor stroma was detectable through gene set analyses.

### Tumor type-dependent characteristics of the KIRC TMEs

Hypoxia and glycolysis-related pathways were significantly upregulated in the KIRC cancer components, whereas these pathways were downregulated in the KIRC stromal components (Figure 3A). Also, the elevated mean enrichment score of endothelial cell markers (Figure 3B) and prominent capillary network formation on microscopic assessments (Figure 3C) were noted in KIRC PDXs. These observations were in line with the well-documented pathophysiology of KIRC that the aberrantly accumulated Hypoxia Inducible Factor 1 (HIF1) induced metabolic shift toward aerobic glycolysis in tumor cells and pro-angiogenic dynamics in the stroma (Cohen and McGovern, 2005) (Gatto et al., 2014). Moreover, given that malignant cells were known to compete for glucose metabolism with stromal cells in the TME (Koukourakis et al., 2006) (Chang et al., 2015), the metabolic competition arising due to their interplay may ascribe complemental regulation of the glycolysis pathway in cancer and stromal cells (Figure 3A).

### Integrated TME analysis to predict paracrine effectors during KIRC homeostasis

As the stromal expression pattern of the KIRC model recapitulated its original tumor well and was unique among other PDX tumor types, we decided to explore the KIRC further.

First, gene-level differential analysis of the stromal component of KIRC against the other eight tumor types revealed 941 differentially expressed (DE) genes (*q*< 0.05, BH adjusted), including the *Mcf2l*, *Exoc3l2*, *Olfr558*, and *Chrm2* (Table S4) listed as the top-ranked genes. Upstream regulators over the top 300 DE gene orthologs (*q*< 0.001) were then estimated using the Ingenuity Upstream Regulator Analysis (IPA) (Krämer et al., 2014) (Figure 1C). Briefly, IPA executed a directional pathway analysis based on the manually curated database of upstream regulators and targeted genes with cause-effect relationships related to expression, transcription, or activation. Specifically, IPA evaluated the extent of overlap between the provided gene set (i.e., top 300 DE gene orthologs of the KIRC stromal component) and the preset downstream genes in the database based on Fisher's exact test. Activation z scores also represented the concordance of expected expression trends and the observed expression trends of downstream genes;

$$z=\frac{\sum_{i}x_{i}}{\sqrt{N}}\;\left(x_{i}\in \left\{-1,1\right\}\right)$$

where$\;x_{i}$ represents the direction of the activation of the $i^{th}$ downstream gene, with i ranging from 1 to *N.* Through this IPA analysis, 246 genes (*p*< 0.05, Fisher's exact test), such as the *DLL4*, *RUNX1*, and *VEGFA* genes were identified as candidate regulators (Figure S2 and Table S5). Note that candidates of stromal regulators listed in Table S5 included any type of upstream factors (e.g., autocrine/paracrine regulators and transcription factors) that were either relevant or irrelevant to cancer-stroma interaction.

Second, the expression uniqueness of stromal regulator homologs in the corresponding cancer cells was evaluated (Figure 1D). Differential analysis on cancer reads of the KIRC against the other 8 PDX types revealed 1,181 DE genes (*q*< 0.05, BH adjusted), with the lowest q-value identified for *POU3F3*, *SLC28A1*, and *CDH6* genes (Table S6).

**Figure 4:**
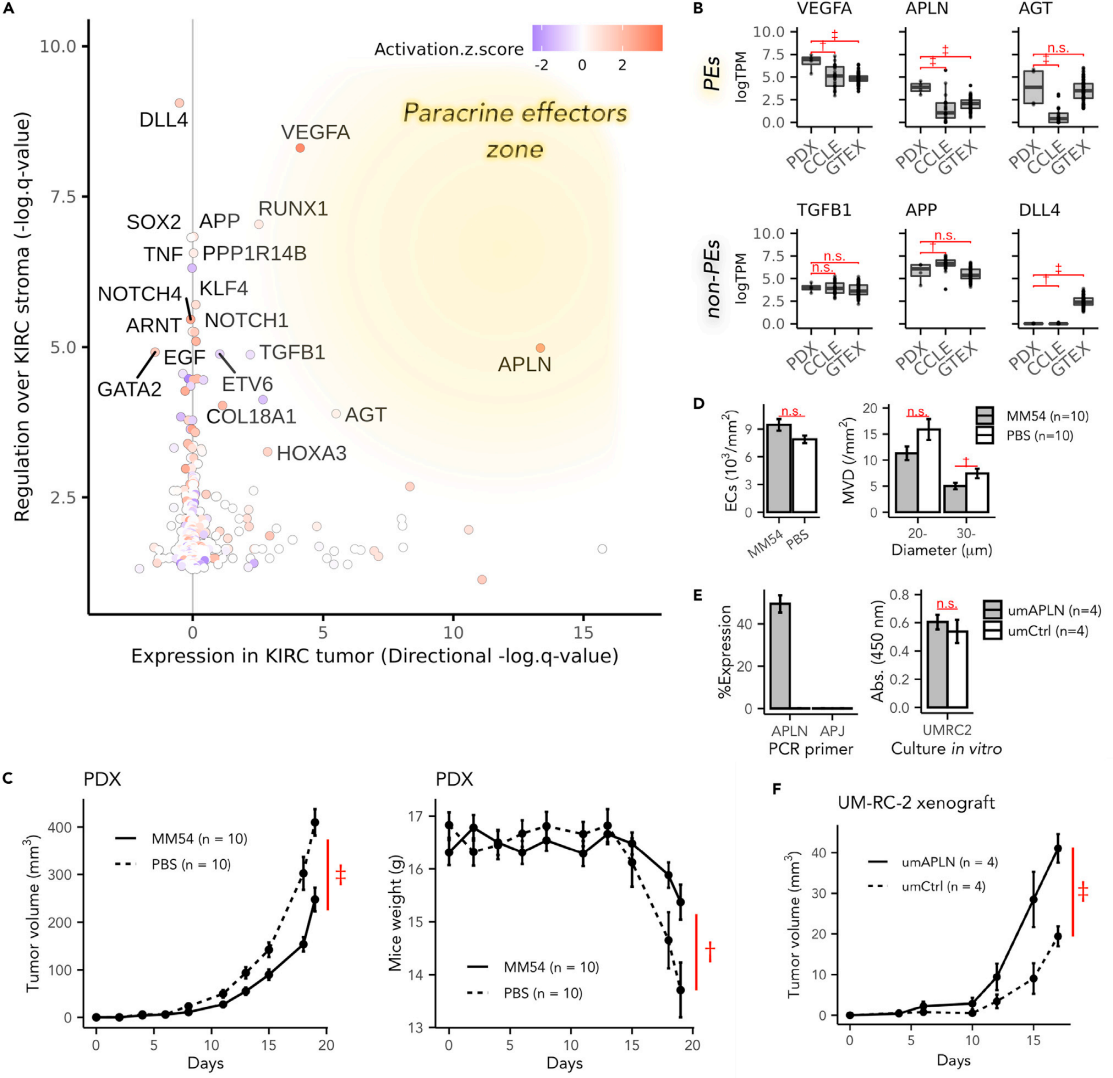
Integrated TME analysis predicted APLN as a paracrine effector during KIRC homeostasis (A) Prediction of the paracrine effectors during cancer-stroma interaction. Xaxis: negative logarithm of the *q*-value (BH adjusted) on the differential expression analysis of KIRC cancer cells. The plus or minus sign was adjusted to represent the direction of gene expression; genes with x>0 were upregulated in KIRC cancer cells and vice versa. Yaxis: negative logarithm of the *q*-value (BH adjusted) of overlap on the upstream regulator analysis of IPA for the top 300 DE genes of the KIRC stroma. The color scale: activation *Z* score indicated the concordance of the predicted direction and the observed direction of downstream genes' expression. (B) Library-size scaled log-expression levels of estimated paracrine regulator genes (*VEGFA*, *APLN*, and *AGT*) and non-paracrine regulator genes (*TGFB1*, *APP*, and *DLL4*) in PDXs, stroma-free KIRC cell lines (CCLE), and cancer-free normal kidney tissue (GTEx). (C) Tumor volume increases, and body weight losses of host mice during the APLN-signaling inhibition test on KIRC PDX mice (MM54-dosed groups, solid lines; PBS-dosed groups, dashed lines). (D) The mean density of endothelial cells (ECs) (the left panel) and mean density of intra-tumor microvessels (MVD) (the right panel) were measured in histopathology sections of KIRC PDXs during the APLN-signaling inhibition test (MM54 groups, shaded bars; PBS groups, white bars). (E) The left panel: Relative expression (% *GAPDH*) of *APLN* and APLN receptors (*APJ*) in *APLN*-overexpressed UM-RC-2 cells (umAPLN, in gray) or control UM-RC-2 cells (umCtrl, in white). The right panel: Absorbance (Abs.) of 450 nm light on CCK8 assay after a 72 h culture for umAPLN (in gray) and umCtrl (in white). (F) Time courses of tumor volumes of umAPLN (solid line) or umCtrl (dashed line) after the subcutaneous transplantation into host mice. (C-F) The data were shown as mean ± standard error of the mean (SEM) (n = 10 or n = 4). The letter n.s.,†, or ‡represents p≧0.05, p<0.05, or p<0.005, respectively, on the two-way Student's t-test (C, F), or the Mann-Whitney U test (B, D, E). See also Figures S2 and S3 and Table S4. DE genes of the KIRC stromal component. Related to, Table S5. Upstream Regulator Analysis of the top 300 DE genes of the KIRC stromal component. Related to, Table S6. DE genes of the KIRC cancer component. Related to Figure 4.

Lastly, genes of steps C and D intersections were retrieved and subsequently integrated into a plot, with regulatory effect scores over the stromal transcriptome plotted on the yaxis (C), and indicators of the expression uniqueness in cancers plotted on the xaxis (D) (Figures 1E and 4A). As a result, the genes in the right-upper area of the plot presented more as paracrine effectors in the cancer-stroma interaction. Notably, the *VEGFA*, *APLN*, and *AGT* genes were more positively expressed in KIRC cancer cells ($q^{VEGFA}=1.7\times 10^{-4}$, $q^{APLN}=1.4\times 10^{-13}$, $q^{AGT}=6.1\times 10^{-7}$), and showed substantial regulatory effects on downstream genes of the stroma ($q.overlap^{VEGFA}=4.9\times 10^{-9}$, $q.overlap^{APLN}=1.0\times 10^{-5}$, $q.overlap^{AGT}=1.3\times 10^{-4}$) in positive directions ($z^{VEGFA}=3.97$, $z^{APLN}=2.83$), although the *AGT* gene showed a relatively weak regulatory trend consistency ($z^{AGT}=0.43$). Therefore, we predicted *VEGFA, APLN,* and *AGT* genes as paracrine effectors for the cancer-stroma interaction in KIRCs, whereas *DLL4, APP*, and *TGFB1* genes to be potential autocrine effectors. It is also important to note that most of the other stroma-regulating genes, such as the *RUNX1*, *PPP1R114B*, and *SOX2*, were either gene coding non-soluble transcription regulators or intracellular proteins. Therefore, they were omitted from the subsequent analyses.

Furthermore, the expression levels of estimated paracrine effectors in immortalized KIRC cell lines (CCLE) and normal kidney tissue of Genotype-Tissue Expression (GTEx) were investigated to evaluate the significance of stromal cells located adjacent to cancer cells (PDX versus CCLE) and potential confounding effects caused by their tissue specificity (PDX versus GTEx), respectively. Interestingly, the relative expression levels of estimated paracrine effectors, such as *VEGFA*, *APLN*, and *AGT,* were found to be strikingly and significantly higher in PDX cancer components than in cell lines obtained from CCLE data (Figure 4B). Also, the *VEGFA* and *APLN* genes in PDXs additionally showed greater expression levels than in normal kidney tissue of GTEx data, suggesting that their expression uniqueness (xaxis in Figure 4A) in KIRC cancer components was not merely attributable to tissue specificity. By contrast, stromal upstream regulators that were not estimated to be paracrine effector genes, such as *DLL4*, *APP*, and *TGFB1*, showed no significant increase in expression levels in KIRC cancer components compared with CCLE or GTEx.

### Validation of the expression profiles unique to KIRC

Here, external data of KIRC PDXs (Wang et al., 2018) (Courtney et al., 2020) were exploratorily incorporated into our original PDX data to confirm the validity of the KIRC homeostasis uniqueness observed in this study so far. The external data included 21 PDXs generated using 17 tumors surgically resected from 15 patients. Results showed that the overall expression patterns (Figure S3A) and expression levels of potential paracrine effectors (Figure S3D) in the external data were comparable to those in our original data. Upregulated hypoxia or glycolytic pathways in cancer components, and downregulated hypoxia or glycolytic pathways in stroma components were significant even when the external data was incorporated (Figure S3B). The endothelial cell signature remained the most dominant in KIRC stroma, although it lost statistical significance (Figure S3B). Furthermore, the log fold-change values of differentially expressed genes (*q*< 0.05) in KIRC based on our original data were highly correlated with the values of corresponding genes in the KIRC of the combined data (Figure S3C). Collectively, KIRC homeostasis observed in our original data seemed robust despite the limited number of samples.

### APLN signaling modulated KIRC tumor progression

To further investigate the role of *APLN* in the KIRC cancer-stromal homeostasis, pharmacological inhibition tests were performed by injecting MM54 (cyclo[1-6]CRPRLC-KH-cyclo[9-14]CRPRLC), an established apelin receptor (APJ)-specific antagonist (Macaluso et al., 2011), intraperitoneally into the KIRC model mice. MM54 has been designed to implement bivalent binding sites “RPRL” (Macaluso et al., 2011), a pharmacophore sequence conserved in human and mouse apelin genes (Yang et al., 2015). The specificity to APJ (Harford-Wright et al., 2017) and the effectiveness in inhibiting APJ-signaling in mouse models (Uribesalgo et al., 2019) have also been shown in previous literatures. The administration of MM54 modestly but statistically significantly suppressed tumor growth by 39% on average, reducing the mean tumor volume from 409.8 mm^3^ in the vehicle-dosed group to 247.5 mm^3^ in the MM54-dosed group (*p*< 0.001) on the 19th day after tumor engraftments (Figure 4C). Furthermore, the density of the intra-tumor microvessels (MVD) (Weidner et al., 1991), having a diameter being larger than 30 μm, was significantly lower in the MM54-administrated group (*p*< 0.05, Figure 4D). However, the total MVD of technically countable vessels was statistically insignificant. Given that the mean density of the endothelial cells (ECs) was equivalent in both groups (Figure 4D), it was suggested that MM54-dosed tumors formed relatively smaller vessels. Meanwhile, Ki67-positive ECs were less than 1% of all ECs in both groups, suggesting that most ECs were not in the mitotic phase (data not shown). To further understand possible mechanisms of how the modulated APLN signaling affected KIRC growth rates, we investigated the effects of *APLN* on cell growth by comparing its results in *in-vitro* and *in vivo* environments. *APLN*-transfected UM-RC-2, a human KIRC cell line with relatively lower endogenous *APLN* expression, showed no significant difference in *in-vitro* cell growth rate (Figure 4E). By contrast, the same cell showed a mild but significant increase in tumor growth rate *in vivo* when it was engrafted in mice (Figure 4F). Besides, the endogenous expression level of the APLN receptor *APJ* was almost negligible in this cell line (Figure 4E). Taken together, these outcomes implied that APLN possessed the pro-carcinogenic effect in the KIRC xenograft model, and that the involvement of stromal cells into tumor tissue was needed for *APLN* to exert its effect.

## Discussion

The systematic analysis of multi-cancer TME was demonstrated to be feasible with the use of PDX models. PDX models recapitulated the original tumor's features, including the cancer transcriptome, relative cancer-stroma ratio, stromal cell composition, and altered cell signaling. An integrated analysis of cancer and stroma transcriptome also detected putative paracrine signaling accelerators, such as the VEGFA, including a less-documented soluble factor, the APLN, in the KIRC pathology. Furthermore, a subsequent APLN inhibition trial on KIRC PDX mice supported its potential function in KIRC tumor progression.

The ESTIMATE stromal scores of the cancer reads were substantially lower than those of the stomal reads (Figure 2A), which supports the validity of the read assignment procedure to mixed human/mouse RNA libraries. It is interesting to note from the results that the stromal scores of cancer components varied across the different PDX types and were almost equivalent to that of the CCLE samples' (Figure 2A). Sarcomas, including the EWS or GIST, except for GBM, showed relatively high stromal scores than those of other PDX types. This result can be attributed to the process of ESTIMATE in the construction of cancer expression references where most of the enrolled tumor types were epithelial cancers, including breast, colon, endometrial, kidney, lung, and ovarian cancers. One exception to the recruitment is the glioma; ESTIMATE included glioma stem-like cell-specific genes into the model (Yoshihara et al., 2013). Furthermore, the high proportion of stromal components in the PAAD and KIRC identified during tumor purity analysis (Figure 2B) was in concordance with the pathological features of original tumors. Primary PAAD is one of the most stroma-rich tumors. Most of the PAAD's stroma comprises fibroblasts and fibrotic ECM, which is a potential obstacle for delivering therapeutic drugs to cancer cells (Feig et al., 2012). The KIRC, meanwhile, induces many intra-tumor vasculatures (Cohen and McGovern, 2005). These TME characteristics unique to tumor types were confirmed in PDXs by the enrichment analysis on cell-type markers (Figure 3B) and during the histological study (Figure 3C). Furthermore, unsupervised pathway analyses captured the well-known KIRC pathophysiology, such as the accumulated HIF1, the accelerated transcription rate of hypoxia-related genes, and the remarkable metabolic shift toward the anaerobic direction (Gatto et al., 2014). The tendency of PDXs to show an overall higher tumor purity than that of the TCGA (Figure 2B) can be explained by the insufficient functionality of immune cells in the host mice, which failed to infiltrate into the tumor. Another possible explanation for the disparity was that the growth rate of implanted tumors surpassed that of the stromal components in the PDXs. Indeed, the host stromal cells should involve a large chunk of the tumor, which had already been 5 mm^3^ in size at the time of the engraftment procedure.

The presented workflow correctly predicted the involvement of the previously documented paracrine regulators, such as VEGFA and AGT, in the KIRC pathological homeostasis. The VEGF-triggered signaling is a well-established pharmacological target for anticancer therapies against metastatic KIRC in clinical use (Motzer et al., 2013). Angiotensin is a vital vasoconstriction factor and a mitogenic factor responsible for inducing tissue remodeling. Upregulated *AGT* expression in KIRC cancer cells could be confounded by the tissue-specificity of its expression in the kidney compared to other organs (Figure 4B). However, it does not necessarily deny the possible role of AGT in cancer-stroma interactions. The aberration of the renin-angiotensin system has been reported as a risk factor for KIRC, and the inhibition of the angiotensin system, combined with VEGF signaling-targeted therapies, improves the prognosis of metastatic KIRCs in several sub-group and retrospective analyses (Derosa et al., 2016). Intriguingly, those paracrine effectors were significantly upregulated in the PDX cancer component compared to the stroma-free cancer cells (Figure 4B). This observation implies that the increased levels of *VEGFA*, *AGT*, and *APLN* genes expression in KIRC are partly due to the stromal involvement in the tumor tissue organization.

Apelin and its innate receptor; APJ, are recently documented axis that is responsible for regulating angiogenic dynamics, fibrotic tissue remodeling, body fluid homeostasis, and adipocyte endocrine secretion (O'Carroll et al., 2013). In our study, the antagonistic inhibition of the APLN signaling led to the growth retardation of KIRC PDXs (Figure 4C). Besides, larger intra-tumor vessels were significantly reduced by administering the APJ-antagonist without increasing the total ECs (Figure 4D). Also, tumor progression induced by *APLN*'s overexpression in UM-RC-2 seemed to depend on the stomal involvement (Figures 4E and 4F). These results are in accord with previously reported roles of apelin, which promoted angiogenesis and vessel enlargement during the process of embryogenesis (Kidoya et al., 2008) and mammary carcinoma progression (Sorli et al., 2007). Regarding the possible mechanism of how APLN influences the vessel diameter, APLN is proposed to induce proliferation and chemotaxis to ECs in the sprouting stage leading to potential mobilization and assembly of vessels (Kidoya et al., 2008). However, this mechanism has not been explicitly proven so far. Collectively, the APLN-APJ axis was proposed to be a druggable target in the cancer-stroma interplay in KIRC. *APLN* transcription is promoted by the HIF1α bound on hypoxia-responsive elements like the *VEGFA* (Eyries et al., 2008), whereas the downstream of APLN signaling is partly independent of VEGFA signaling (Wu et al., 2017). Therefore, given that both the VEGFA and APLN exerted angioplasty effects (Figures 3B and 3C) on the stroma in a paracrine way (Figures 4A and 4B), the hypoxia-related signaling initiated by the aberrantly accumulated HIF1 in the KIRC tumor can be acknowledged as their shared upstream origin (Figure 3A).

The fact that the PDX host mice lack functional lymphocytes or natural killer cells in the TME constitution (Ito et al., 2002) should be noticed when the results in this study are interpreted. Those immune cells provide complexity and dynamics to the TME due to their heterogeneity and motility. Therefore, the absence of immune cells in the PDX model leads to a disadvantage in ignoring the effects of immunological dynamics in the TME. At the same time, however, it also provides the model with a unique advantage to make the interplay of cancer cells and the less motile stroma (fibroblasts or vessel components) easier to understand by reducing the TME complexity. The use of humanized mouse models in which hematopoietic stem cells or peripheral blood mononuclear cells are engrafted is proposed to assist with reproducing more precise TMEs. Regarding combination therapies assessed in *in-vivo*, overexpressed *APLN* was reported to promote the maturation of the cancer vasculature and subsequently enhanced its immune therapy efficiency in orthotopic colon cancer models (Kidoya et al., 2012). In contrast, the additional anticancer effect of multi-target tyrosine kinase inhibitors against metastatic KIRC has also been proven in clinical studies along with IFNα or the mammalian target of rapamycin (mTOR) inhibitors (Choueiri and Motzer, 2017). It would thus be worthy of examining whether the APLN-signaling inhibition helps with improving chemotherapy responses in clinical settings as well.

Several available alternatives to predict the tumor-stroma crosstalk instead of IPA exist. For example, Choi et al. developed a Cell-Cell Communication Explorer (CCCExplorer), which is a computational model of a crosstalk network composed of ligand-receptor sets (intercellular signaling), transcriptional factors, and their targets (intracellular signaling) (Choi et al., 2015). The CCCExplorer is superior in that the model details transcriptional processes so that its outputs are straightforward to interpret in biological contexts. In contrast, IPA includes all gene sets in cause-result relationships involved in various transcriptional processes driven by transcriptional factors and enhancer-binding or histone modification; therefore, the analysis can be more comprehensive.

Previous studies have confirmed the importance of the cross-cancer TME analysis by employing the scRNASeq approach (Qian et al., 2020). This study integrated publicly available scRNASeq data to map each cancer in terms of their stromal constitutions, which led to an understanding of the tumor-associated stromal pathophysiology of melanomas. Currently, only a few publicly available PDX data exist. Still, efforts are being made to organize published PDX data for researchers to easily access these data via data portal sites, such as the PDX Finder (Conte et al., 2019). So far, relatively little progress has been made on the characterization of TMEs in analyses that enroll multiple PDX tumor types (Bradford et al., 2016). Therefore, the multi-cancer analysis of larger-scale PDX data is believed to broaden the perspective regarding the biology of malignancies, especially in cancer-stroma communications, and pave the way to discover novel anticancer drug targets that will shoot the Achilles tendon of cancer-stromal interactions.

### Limitations of the study

One of the major limitations in this study is that the sample size of each tumor cohort was relatively small; thus, the power to detect tumor-type-dependent crosstalk can be diminished. Therefore, it would be preferable to verify the results by analyzing more extensive PDX data comprising more samples per tumor type in future researches. Also, the cluster formation in the t-SNE plot was inadequate in some tumor types, particularly in NSCLC (Figure 2A). A possible reason was that NSCLC included various histological subtypes, but the non-biological reasons, such as technical limitations, cannot be excluded due to the scarcity of the samples. In addition, cancer-stoma communications in PDX models were intrinsically limited by ligand-receptor interactions that were conserved across species, potentially leading to the unnatural remodeling of intra- or inter-cellular signaling. Other than the above mentioned, it was unclear on what process MM54 worked, tumor engraftment or tumor progression, because MM54 was administrated from the day of tumor engraftment.

## STAR★Methods

### Key resources table

**Table:** 

REAGENT or RESOURCE	SOURCE	IDENTIFIER
**Antibodies**		

Anti-CD31 antibody	Abcam	Cat#ab28364; RRID:AB_726362

**Biological samples**		

Patient-derived xenografts (PDX)	This study	N/A
Chemicals, Peptides, and Recombinant Proteins		
MM54	Tocris Bioscience	CAS 1313027-43-8

**Critical commercial assays**		

Cell Counting Kit-8	DOJINDO	Cat#CK04
PikoReal™Real-Time PCR System	Thermo Fisher Scientific	N/A

**Deposited data**		

Human transcript sequences	The GENCODE Project	https://www.gencodegenes.org/human/release_27.html; RRID:SCR_014966
Mouse transcript sequences	The GENCODE Project	https://www.gencodegenes.org/mouse/release_M15.html; RRID:SCR_014966
HomoloGene build 68	(NCBI Resource Coordinators, 2016)	https://ftp.ncbi.nih.gov/pub/HomoloGene/build68/; RRID:SCR_002924
Molecular Signatures Database v6.2	(Liberzon et al., 2015)	https://www.gsea-msigdb.org/gsea/msigdb/; RRID:SCR_016863
Cancer Cell Line Encyclopedia	(Ghandi et al., 2019)	https://ocg.cancer.gov/ctd2-data-project/translational-genomics-research-institute-quantified-cancer-cell-line-encyclopedia; RRID:SCR_013836
Genotype-Tissue Expression	GTEx Consortium	https://www.gtexportal.org/home/datasets; RRID:SCR_013042
Sequence data of KIRC PDXs	(Wang et al., 2018)	EGA: EGAD00001003895
Sequence data of KIRC PDXs	(Courtney et al., 2020)	EGA: EGAD00001004799
Expression data of human and mouse genes in PDXs	This study	GEO: GSE159702
Tumor purity estimates of The Cancer Genome Atlas samples	(Aran et al., 2015)	https://gdc.cancer.gov/about-data/publications/pancanatlas

**Experimental models: Cell lines**		

UM-RC-2	ECACC	Cat#08090511; RRID:CVCL_2739

**Experimental models: Organisms/strains**		

Mouse: NOD.Cg-*Prkdc*^*scid*^*Il2rg*^*tm1Sug*^/ShiJic (NOD/Shi-scid,IL-2RγKO)	In-Vivo Science	RRID:MGI:6197549

**Oligonucleotides**		

Human *APJ* primer, forward (CCCCTTCCTCTATGCCTTTTTC)	Eurofins Genomics	N/A
Human *APJ* primer, reverse (ATCTGTTCTCCACCCTTGCC)	Eurofins Genomics	N/A
Human *APLN* primer, forward (ACCCAAG CTGGCTAGTTAAGCCACCATGAATCT GCGGCTCTGCGTGCA)	Eurofins Genomics	N/A
Human *APLN* primer, reverse (TGTTCGAA GGGCCCTCTAGATCAGAAAG GCATGGGTCCCT)	Eurofins Genomics	N/A

**Software and algorithms**		

Salmon version 0.8.1	(Patro et al., 2017)	RRID:SCR_017036
ESTIMATE version 1.0.13	(Yoshihara et al., 2013)	https://bioinformatics.mdanderson.org/estimate/
GSVA version 1.36.0	(Hänzelmann et al., 2013)	RRID:SCR_021058
Ingenuity Pathway Analysis version 01–07	QIAGEN	RRID:SCR_008653
Olympus cellSens Software	Olympus Corporation	RRID:SCR_014551
R version 4.0.0	https://www.r-project.org/	RRID:SCR_001905

**Other**		

Resource website for codes and supplemental tables	This study	https://github.com/Kuniyo-Sueyoshi/PDX/

### Resource availability

#### Lead contact

Further information and requests for resources and reagents should be directed to and will be fulfilled by the lead contact, Shumpei Ishikawa (ishum-prm@m.u-tokyo.ac.jp).

#### Materials availability

This study did not generate new unique reagents.

### Experimental model and subject details

#### Mice

Five- to six-week-old female NOD.Cg-*Prkdc*^*scid*^*Il2rg*^*tm1Sug*^/ShiJic (NOD/Shi-scid, IL-2RγKO) mice (RRID: MGI:6197549, In-Vivo Science, Tokyo, Japan) (Ito et al., 2002) were purchased to establish PDXs as described in the METHOD DETAILS section. Sample size (n) was mentioned in each figure legend. Experiments using human xenografts and mice were also conducted following the animal experiment guidelines of the University of Tokyo and the Tokyo Medical and Dental University.

#### Human tumor samples

Tumors surgically resected from 43 patients at the Kanagawa cancer center were used to establish PDXs. Basic characteristics of patients such as age and sex were provided in Table S1 if available. The age and sex of 12 patients were unavailable because of technical issues. The ethics board of the University of Tokyo approved this study. Written informed consent was obtained from all patients. The statement in the consent documents included studying resected specimens and disclosing data at any publicly accessible repositories in such a form that individuals cannot be specified.

#### Cell line

A human renal cell carcinoma cell line, UM-RC-2 cell (RRID: CVCL_2739, ECACC 08090511), was obtained from the European Collection of Authenticated Cell Cultures (ECACC). Cells were cultured in the EMEM medium (FUJIFILM Wako Pure Chemical Corporation, Japan) and supplemented with 10% fetal bovine serum (#172012, Sigma Aldrich, MO, USA), sodium pyruvate (#11360-070, Thermo Fisher Scientific, MA, USA), penicillin/streptomycin (#168-23191, FUJIFILM Wako Pure Chemical Corporation), NEAA (#11140050, Thermo Fisher Scientific), and L-Glutamine (#073-05391, FUJIFILM Wako Pure Chemical Corporation) at 37°C under a condition of 5% CO_2_.

### Method details

#### Model mice establishment and RNA sequencing

Forty-three fresh tumor specimens of 9 distinctive tumor types (Table S1) were resected at the Kanagawa cancer center, sectioned, and frozen at −80°C. Dissolved tumor fragments of 5 mm^3^ in size were subcutaneously transplanted into the flank of NOD/Shi-scid, IL-2RγKO mice (In-Vivo Science, Japan) (Ito et al., 2002) to establish PDXs. PDX samples were re-transplanted to reproduce the$\;N^{th}$ passages (N: 2, 3, …) of the corresponding PDX, yielding 70 PDX samples (Table S1). PDXs of the 2^nd^ or later passages were enrolled in the following analyses. Subsequently, model mice were euthanized, and tumors were resected for RNA extraction. Each sample passed the inspection for syphilis, hepatitis B virus, hepatitis C virus, or HIV infection. Total RNA of the tumors, suspended in TRIzol Reagent (Thermo Fisher Scientific), was extracted according to the manufacturer’s instruction. Then, the sequencing library was prepared using 1 mcg of total RNA as the starting material of TruSeq Stranded mRNA Library Preparation kit (Illumina, San Diego, CA, USA). Briefly, PolyA+ RNA purified from total RNA was fragmented using divalent cations. Double-stranded cDNA was synthesized using Invitrogen SuperScript II Reverse Transcriptase (Thermo Fisher Scientific), then the indexed RNA adapter was ligated. The cDNA fragment was amplified by PCR as following; denatured for 30 s at 98°C; cycled for 15 times of 10 s at 98°C, 30 s at 60°C, and 30 s at 72°C; and incubated for 5 min at 72°C for final extension, then cooled to 4°C. The amplified library was purified by Agencourt AMPure XP (Beckman Coulter, Tokyo, Japan). Libraries were sequenced 100bp paired-end on the Hiseq2000 sequencer (Illumina). Four libraries were loaded into a single lane of Illumina flow cell, producing more than 30 million paired-end reads for each sample.

#### Assignment and quantification of transcripts

The Salmon (version 0.8.1) software, which is aware of biases derived from the fragment length, distance from the 5′ and 3′ end of the sequenced fragments, and regional GC content (Patro et al., 2017), was used for transcript quantification. Briefly, the human (GENCODE, release 27, GRCh38.p10) and mouse transcriptomes (GENCODE, release M15, GRCm38.p5) were combined, and a quasi-mapping index of the xeno-species transcriptome was subsequently built using the *--type quasi -k 31* preset. Then, the transcripts of the protein-coding regions were extracted from the whole transcripts. Estimation of transcript counts and abundance was also executed using the Salmon quasi-mapping mode with the *–l A* preset. Here, yielded data were divided into human or mouse transcripts, representing cancer reads or stroma reads, respectively. Subsequently, the transcript-level count estimates and abundance were summarized to gene-level length-scaled count estimates and abundance in transcript per million (TPM) values with the *tximport* package (version 1.16.1) (Soneson et al., 2015) in R (version 4.0.0) using the *summarizeToGene* function with the *countsFromAbundance = “lengthScaledTPM”* preset on the reference of the transcriptome index prepared above. Genes with constantly low expression were filtered off then used in the t-SNE and differential expression analysis below. The cutoff criteria for filtering was whether TMM (Trimmed Mean of M value)-normalized CPM (Count Per Million) values were constantly lesser than one. These values were computed using the *edgeR* package (version 3.34.0) (Robinson and Oshlack, 2010). The base-2 logarithm of CPM values with a pseudo-count of 0.25 was used in most subsequent studies unless otherwise noted. The data of transcript-level abundance of the mixed transcripts and the gene-level expression matrices are available at the Gene Expression Omnibus (GEO) site. (See the data and code availability section).

#### Overview of the PDX transcriptomes

Dimension reduction by the t-SNE algorithm was conducted using the R package *Rtsne* (version 0.15), with perplexity set to five. Stromal scores of PDXs and CCLE expression data in log-TPM values were calculated using the ESTIMATE (Estimation of STromal and Immune cells in MAlignant Cancer tissues using Expression data, version 1.0.13), according to the instructions of the developer (Yoshihara et al., 2013). The CCLE data of corresponding primary tumors were also retrieved from the NIH site; https://ocg.cancer.gov/ctd2-data-project/translational-genomics-research-institute-quantified-cancer-cell-line-encyclopedia (accession date; May 29, 2020). Subsequently, we defined the tumor purity of a PDX sample as the proportion of the gene-level and length-scaled count sum of the cancer components to that of the total (cancer plus stroma) components. Tumor purity estimates of TCGA computed by the ABSOLUTE algorithm, which infers the relative abundance of malignant cells based on their allelic copy number profiles, were also retrieved from a previous study (Aran et al., 2015). Furthermore, mouse gene symbols were converted to human gene symbols using the HomoloGene database (build 68) (NCBI Resource Coordinators, 2016) as needed.

#### Gene set analysis

Gene Set Variation Analysis (GSVA, version 1.36.0) (Hänzelmann et al., 2013) was conducted for pathway analyses and a stromal cell-signature analysis. GSVA is an unsupervised, competitive gene set analysis approach that gives a sample-wise enrichment score for gene sets by simulating the Kolmogorov-Smirnov-like random walk. For the pathway analyses, 40 pathways of 50 curated Hallmark pathways (Table S2) were obtained from the Molecular Signatures Database (MSigDB v6.2, Broad Institute, Boston, USA) (Liberzon et al., 2015). Ten pathways considered irrelevant to the TME pathophysiology were excluded in advance because this analysis's primary motivation was to describe stromal expression profiles. For the cell-signature analysis, eight stromal cell types and gene sets (Table S3) were also prepared in reference to previous studies (Schelker et al., 2017) (Yu et al., 2019) (Şenbabaoğlu et al., 2016). The statistical method of PDX type-wise differential analyses of the enrichment scores was described in the section below.

#### Differential expression analysis of PDXs

The Limma-voom pipeline (Law et al., 2014) (Smyth, 2004) was used for the RNA-seq differential analysis on the stroma and cancer of the KIRC PDX model. The sample-wise mean-variance relationship was stabilized using the *voom* function to fit the RNA-seq data to the negative binomial distribution. A linear model to explain the expression level of the gene was also created as a function of cancer type, using the R package; *limma* (version 3.48.0), and the linear model as shown below

(Equation 1) $$\begin{array}{l} Y=\beta_{0}+D\beta +\varepsilon_{0}\; \end{array}$$

whereY is a vector of the *voom*-stabilized transcript abundance of the gene, $\beta_{0\;}$is the intercept, βis a vector of coefficient, and $\varepsilon_{0}\;$is a residual vector. The design matrix $D_{70\times 9}$ was set as

$$D=\;\left[\begin{array}{lllll} D_{1} & O & O & O & O\\ O & D_{2} & O & O & O\\ O & O & \ldots & O & O\\ O & O & O & D_{8} & O\\ O & O & O & O & D_{9} \end{array}\right]$$

where$\;D_{i}$ is a $1\times n_{i\;}$matrix, *i* represents each PDX cancer type (i=1,2,⋯,9), and $n_{i}\;$is the total number of samples of the *i*^th^ PDX cancer type.

$${\text{D}}_{i}={\left(1,\;1,\;\ldots ,\;1\right)}^{tr}$$

$$\sum_{i}n_{i}=70$$

PDX samples of multiple passages were also taken as “technical duplicates” to make the most of the gene-wise variance information within each PDX type. The duplicate correlation coefficients of the expression among passages, computed by the *duplicateCorrelation* function, were incorporated as *block* presets in the linear model. Given that a PDX type corresponds to the case of i=I, the contrast matrix $C_{1\times 9}$ was set as below to clarify the PDX type (i=I) vs. others on differential analyses, assuming that samples of the PDX type (i=I) were an experimental group, whereas the remaining eight types were the control group.

$$C_{1\times 9}={\left(c_{1},c_{2},\;\ldots ,c_{9}\right)}^{tr}$$

$$c_{i}=\left\{ \begin{array}{l} \;1\;\left(i=I\right)\\ -1/8\;\left(i\neq I\right) \end{array}\right.$$

Lastly, moderated t-statistics were calculated using the empirical Bayes (eBayes) algorithm that yields a fold change of expression level and *p*-value for each gene. Genes with a *p*-value adjusted by the BH method being less than 0.05 were defined as DE genes (Tables S4 andS6).

#### Differential analysis in gene set analyses

PDX type-wise differential analyses of the enrichment scores in the pathway analysis or cell type signature analysis were conducted using linear models similar to those described in the section “Differential expression analysis,” except for the *voom* step. When modeling the Equation (1), Y was defined as a vector of normalized enrichment scores of a pathway or cell type, while other parameters remained the same as described above. The subsequent processes were also similar to those described above.

#### Estimation of paracrine effectors in KIRC TME

The top 300 DE genes of the stromal component of PDXs were converted to human gene symbols based on the HomoloGene database (NCBI Resource Coordinators, 2016). The *p*-values and log-fold change values of 300 DE genes were analyzed using the Ingenuity Upstream Regulator Analysis tool (Krämer et al., 2014) in the Ingenuity Pathway Analysis (IPA®, version 01–07, QIAGEN Inc.,https://www.qiagenbioinformatics.com/products/ingenuity-pathway-analysis) to estimate the upstream regulators over the stromal transcriptome (Table S5 and Figure S2). Paracrine effectors in cancer to stroma communications were assessed as described in the Results section. The expression data of the KIRC immortalized cell line were also obtained from the CCLE database as described above. Furthermore, expression data of cancer-free kidney tissue data were downloaded from the Genotype-Tissue Expression (GTEx) portal; https://www.gtexportal.org/home/datasets, using the dbGaP accession number phs000424.v8.p2 on 8/27/2019. GTEx Project was supported by the Common Fund of the Office of the Director of the National Institutes of Health, and by NCI, NHGRI, NHLBI, NIDA, NIMH, and NINDS.

#### Validation of KIRC PDXs' expression profiles

Sequence data of KIRC PDXs were obtained from the European Genome-Phenome Archive using the study ID; EGAD00001003895 (Wang et al., 2018) and EGAD00001004799 (Courtney et al., 2020), with permission from the corresponding Data Access Committee. The same methods were also applied to yield and explore expression data described above. Because the data EGAD00001004799 included seven samples from three unique tumor specimens exploited from one patient’s different tumor sites, the number of unique KIRC PDXs was taken as three with several passages when incorporating passage information into a linear model during differential analyses.

#### Apelin receptor inhibition test

A dissolved 4^th^ passage PDX specimen of the KIRC metastasized to the skin was xenografted in the flank of NOD/Shi-scid,IL-2RγKO mice. Subsequently, the mice were raised until the tumor grew to approximately 1cm in diameter and exploited for the following apelin receptor inhibition experiment. The specimens were cut in 1mm cubic fragments, then xenografted in 20 mice subcutaneously. Six mg/kg MM54 (Tocris Bioscience, Bristol, UK) or PBS was then administered intraperitoneally to each ten host mice tri-weekly starting from the day of the tumor transplantation. On every intervention day, the body weight and tumor size were measured. The tumor volume was calculated assuming the tumor shape to be a spheroid;

$$V=\frac{4}{3}\pi ab^{2}$$

Where a denotes the major radius, and b represents a minor radius of the tumor.

#### Pathohistological analysis

Samples were fixed with formalin, embedded in paraffin, then sectioned at 5 μm thickness for pathological evaluation. Details are described in a previous study (Komura et al., 2016). ECs were subsequently stained with anti-CD31 antibody (Rabbit polyclonal anti-CD31 antibody, ab28364, Abcam plc, Cambridge, UK). Histological images of the tumor sections’ whole area were also captured using a digital image analysis system (NanoZoomer-Digital-Pathology apparatus, Hamamatsu Photonics, Japan) for the subsequent microvessel density (MVD) analysis. The microvessel diameter was defined as the minor axis length of the intra-CD31-positive-vascular area that allows a maximum-inscribed ellipse inside. All the intra-tumor vessels with their diameters being larger than 20 μm were also counted across the whole area of a tumor section. However, blood vessels with diameters smaller than 20 μm were not counted because the microvessel network was so highly developed in some tumor areas like “a web” that counting such small vessels was technically challenging. ECs were manually counted on ten images of 400 magnified fields randomly selected out of a tumor slide. The counts were subsequently summed up for each PDX tumor sample using the cellSens® Standard software (Olympus Corporation, Tokyo, Japan) and later averaged across 10 PDX mice.

#### *APLN*cDNA overexpression in UM-RC-2 cells

A human renal cell carcinoma cell line, UM-RC-2 cell (ECACC 08090511), was obtained and cultured as described in the Experimental Model And Subject Details section. Full-length cDNA fragments of the human *APLN* gene were inserted in pcDNA6-myc/His A plasmids (Thermo Fisher Scientific) using the NEbuilder system (E5520, New England Biolabs, MA, USA) to generate human *APLN*-overexpressed UM-RC-2 cells. Sanger sequencing was then used to confirm this inserted sequence. The plasmid containing human *APLN* cDNA and the corresponding empty vector was transfected as well into UM-RC-2 cells using FuGene HD (E2311, Promega, WI, USA) according to the manufacture’s protocol. 48 h after the transfection, Blasticidin (R21001, Thermo Fisher Scientific) was added to the culture medium for the drug selection of transfected cells.

#### Quantitative real-time PCR(qPCR)

Total RNA was extracted from cells using the RNeasy mini kit (#74104, Qiagen, Germany), after which cDNA was synthesized using the SuperScript™ III First-Strand Synthesis System (#18-080-051, Thermo Fisher Scientific). Then, THUNDERBIRD® SYBR qPCR Mix (TOYOBO, Osaka, Japan) was used with PikoReal™ Real-Time PCR System (Thermo Fisher Scientific), all of which were performed according to the manufacturer’s protocols. The following PCR primers (Eurofins Genomics, Tokyo, Japan) were used: human *GAPDH* (Forward 5′-TTGCCATCAATGACCCCTTCA-3′ and Reverse 5′-CGCCCCACTTGATTTTGGA-3′), human *APLN* (Forward 5′-ACCCAAGCTGGCTAGTTAAGCCACCATGAATCTGCGGCTCTGCGTGCA-3′ and Reverse 5′-TGTTCGAAGGGCCCTCTAGATCAGAAAGGCATGGGTCCCT-3′) including adjacent plasmid sequences of the inserted locations, and human *APJ* (Forward 5′-CCCCTTCCTCTATGCCTTTTTC-3′ and Reverse 5′-ATCTGTTCTCCACCCTTGCC-3′). Relative mRNA expression levels of *APLN* and *APJ* compared to *GAPDH* were subsequently calculated by the ΔCt method.

#### *In vitro* and *in vivo* cell growth assay

UM-RC-2 cells transfected with either human *APLN* cDNA or empty vectors were harvested, and 8.0x10^3^ cells were plated in 96-well plates in tetraplicates. After a 72-h cell culture, the Cell Counting Kit-8 (CK04, DOJINDO, Japan) assay was performed according to the manufacturer's protocol, and absorbance at 450nm was measured using EnSpireTM (PerkinElmer, MA, USA). For *in vivo* tumor growth assay, the UM-RC-2 cells transfected with either human *APLN* cDNA or the empty vector were harvested and subcutaneously injected into the skin of NOD/Shi-scid, IL-2RγKO Jic (NOG) mice (2.25x10^6^ cells per mouse) (n = 4) (In-Vivo Science, Japan). The same formula was also applied for calculating the tumor volume as described above.

### Quantification and statistical analysis

Statistical analyses were conducted using R version 4.0.0 (RRID: SCR_001905). In two-group comparison analyses, the two-way Student's t-test was applied to compute pvalues when observations were assumed to be normally distributed and equally variance (Figures 4C and 4F); otherwise, Mann-Whitney U tests were employed (Figures 4B, 4D, and 4E). In the analysis of variance to select the top 10 notable pathways in the stroma (Figure 3A), the one-way ANOVA test was conducted on each 70 enrichment scores across nine PDX tumor groups. The statistical descriptions about differential analyses of PDX transcriptomes and gene-set enrichment scores were detailed in the Method Details section. Benjamini-Hochberg (BH) procedures were employed to control the false discovery rate in multiple comparison analyses, yielding q-values. The difference was considered to be significant when p- or q-value <0.05.

## Data Availability

• The data of transcript-level abundance of the mixed transcripts of each PDX sample and the gene-level expression matrices in count estimate values of cancer and stromal components are available at the GEO (accession number: GSE159702). • Codes used to obtain the expression data from the sequencing data (fastq), result figures, and supplementary tables are available at https://github.com/Kuniyo-Sueyoshi/PDX. • Any additional information required to reanalyze the data reported in this paper is available from the lead contact upon request.

## Supplemental information

Document S1. Figures S1–S3

Table S1. PDX sample list, related to Figure 2

Table S2. GSVA enrichment scores of Hallmark pathways, related to Figure 3

Table S3. Cell type-specific genes used in the geneset enrichment analysis, related to Figure 3

Table S4. DE genes of the KIRC stromal component. Related to Figure 4

Table S5. Upstream Regulator Analysis of the top 300 DE genes of the KIRC stromal component. Related to Figure 4

Table S6. DE genes of the KIRC cancer component. Related to Figure 4 Supplementary tables are also available at https://github.com/Kuniyo-Sueyoshi/PDX.
