# Supplementary material for: Multi-tumor analysis of cancer-stroma interactomes of patient-derived xenografts unveils the unique homeostatic process in renal cell carcinomas
Source: iScience. 2021 Oct 21;24(11):103322. doi: 10.1016/j.isci.2021.103322 (PMC8767947; doi:10.1016/j.isci.2021.103322)
Supplement: Document S1. Figures S1–S3 [file mmc1.pdf]

iScience, Volume *24*

Supplemental information

Multi-tumor analysis of cancer-stroma interactomes of patient-derived xenografts unveils the unique homeostatic process in renal cell carcinomas

Kuniyo Sueyoshi, Daisuke Komura, Hiroto Katoh, Asami Yamamoto, Takumi Onoyama, Tsuyoshi Chijiwa, Takayuki Isagawa, Mariko Tanaka, Hiroshi Suemizu, Masato Nakamura, Yohei Miyagi, Hiroyuki Aburatani, and Shumpei Ishikawa

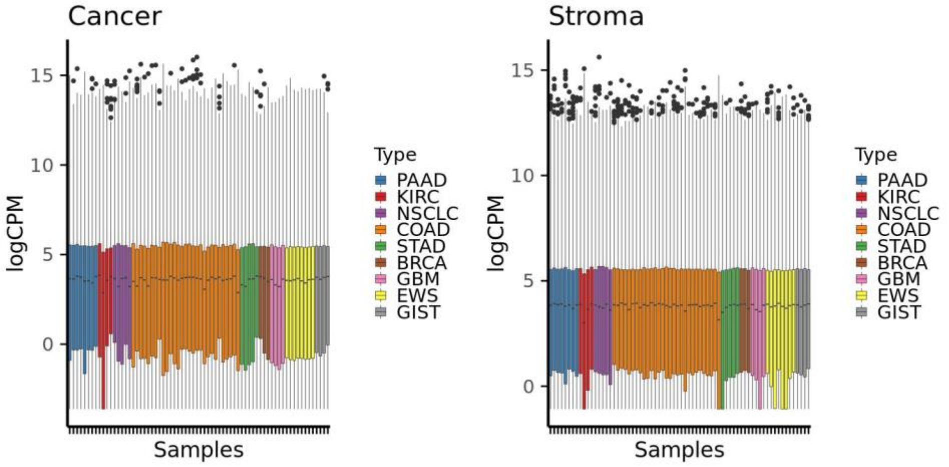

Figure S1. Gene expression distribution of cancer and stromal components of PDXs, Related to Figure 2.

The y-axis "logTPM" represents the logarithm of TMM-normalized TPM values, with the prior count being 0.25. A whisker in each box indicates the median logTPM value of a sample. The lower limit of each box represents the first quartile, whereas the upper limit does the third quartile.

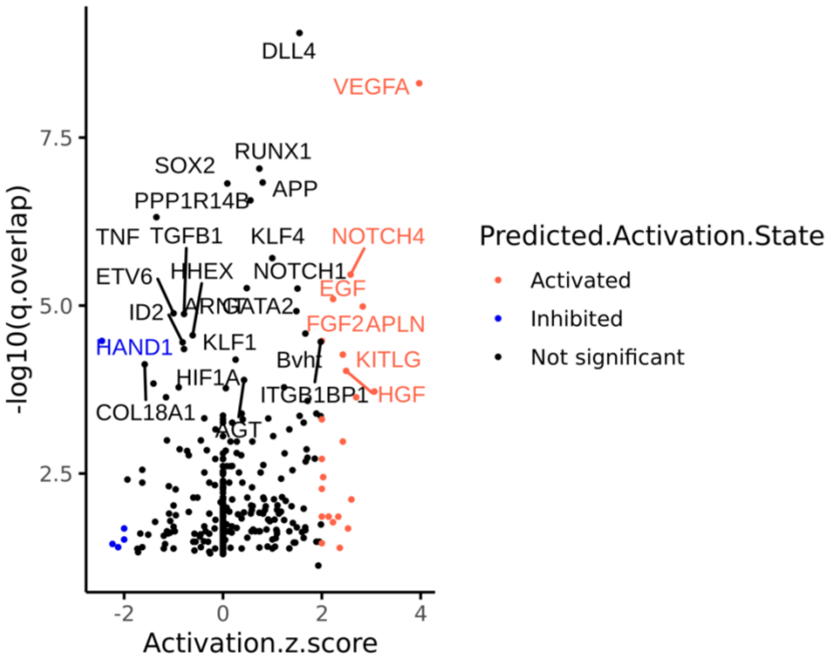

Figure S2. Estimated upstream regulators over the top 300 DE genes in KIRC stroma, Related to Figure 4.

X-axis: statistics of the activation *z*-score that indicate the concordance of the predicted direction and the observed direction of the expression of the preset downstream genes. $z = \frac{\sum_i x_i}{\sqrt{N}} \ (x_i \in \{-1,1\})$, where x_i represents the direction of the activation of ith downstream gene, with i ranging from 1 to *N.* Upstream genes are defined as significantly activated (red) or inhibited (blue) if the absolute value of the z score is larger than 2. Y-axis: negative logarithm of the *p*-value of the overlap on the Fisher's exact test for the preset downstream genes (IPA®) as well as the observed genes in the top 300 DE genes of KIRC stroma.

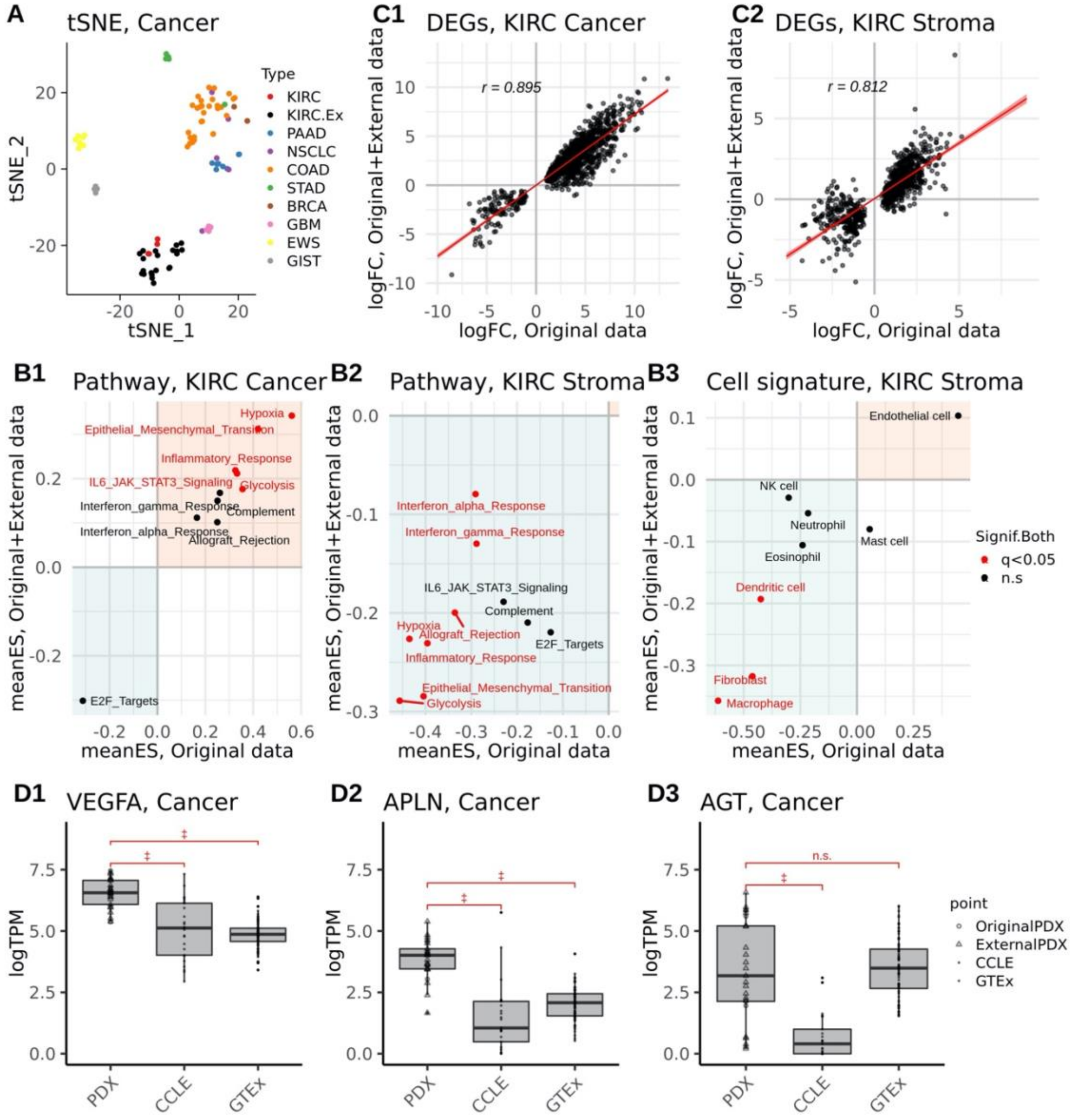

Figure S3. **The concordance of our original KIRC data with external data, Related to Figure 4.**

Our original KIRC data consist of 4 PDXs from 2 patients, while external data include 21 PDXs from 17 tumor sites of 15 patients. **(A)** The t-SNE plot of the cancer components of the external data (dots in black) and our original PDXs (colored dots). **(B)** Mean enrichment scores (meanES) of pathway analyses **(B1, B2)** and a cell signature analysis **(B3)** with or without the external KIRC samples. Dots and labels highlighted in red indicate pathways or cell types of q < 0.05 in the original data, and q < 0.05 in the combined data on the moderated t-test. **(C)** Log fold-change (logFC) of differentially expressed genes identified in our original KIRC data were shown. Pearson's correlation coefficients *r* and linear regression lines with SEM ranges are shown in the plots. **(D)** The relative expression levels of estimated paracrine

effectors; VEGFA **(D1)**, APLN **(D2)**, and AGT **(D3)** in PDX cancer components, KIRC cell lines (CCLE), and normal kidney tissue samples (GTEx). The letter n.s., †, or ‡ in red represent $p \geqq 0.05$, $p < 0.05$, or $p < 0.005$ on the Mann–Whitney U test, respectively.
